# Integrating GDF-15 into Multimarker Assessment of Acute Heart Failure: Diagnostic and Prognostic Implications

**DOI:** 10.3390/life16030503

**Published:** 2026-03-19

**Authors:** Bianca-Ana Dmour, Minerva Codruta Badescu, Daniela Constantinescu, Cristina Tuchiluș, Corina Maria Cianga, Gina Eosefina Botnariu, Ionela Lăcrămioara Șerban, Awad Dmour, Amelian Madalin Bobu, Alexandru Dan Costache, Maria-Ruxandra Cepoi, Sandu Cucută, Irina Iuliana Costache-Enache

**Affiliations:** 1 Grigore T. Popa University of Medicine and Pharmacy Iasi, 700115 Iasi, Romaniaamelian.bobu@gmail.com (A.M.B.); cepoi_maria-ruxandra@d.umfiasi.ro (M.-R.C.);; 2 Cardiology Clinic, “St. Spiridon” County Clinical Emergency Hospital, 700111 Iasi, Romania; 3 III Internal Medicine Clinic, “St. Spiridon” County Clinical Emergency Hospital, 700111 Iasi, Romania; 4 Immunology Laboratory, “St. Spiridon” County Clinical Emergency Hospital, 700111 Iasi, Romania; 5 Microbiology Laboratory, “St. Spiridon” County Clinical Emergency Hospital, 700111 Iasi, Romania; 6 Clinical Center of Diabetes, Nutrition and Metabolic Diseases, “St. Spiridon” County Clinical Emergency Hospital, 700111 Iasi, Romania; 7 Department of Orthopaedics and Traumatology, “St. Spiridon” County Clinical Emergency Hospital, 700111 Iasi, Romania; 8 Clinical Rehabilitation Hospital, 700661 Iasi, Romania

**Keywords:** acute heart failure, GDF-15, biomarkers, multimarker strategy, diagnosis, prognosis

## Abstract

Acute heart failure (AHF) is a leading cause of hospitalization and mortality worldwide. Despite advances in biomarker-based evaluation, accurate diagnostic and prognostic stratification remains challenging in everyday clinical practice. Growth differentiation factor-15 (GDF-15) has emerged as a biomarker associated with advanced disease profiles, poor outcomes and complex underlying pathophysiological processes in heart failure (HF). This study aimed to investigate the diagnostic and prognostic value of GDF-15 in the acute setting and to evaluate its incremental role within multimarker assessment strategies. In this prospective cohort study, 60 patients hospitalized with AHF and 42 control subjects were enrolled. Circulating levels of GDF-15, N-terminal pro–B-type natriuretic peptide (NT-proBNP), and high-sensitivity cardiac troponin I (hs-cTnI) were measured at admission. Diagnostic performance was assessed using receiver operating characteristic (ROC) analysis and multivariable logistic regression. Prognostic value was evaluated for in-hospital, short-term, and one-year mortality, including multimarker models. GDF-15 levels were significantly elevated in AHF and demonstrated a favorable diagnostic profile, with high specificity and acceptable sensitivity. When integrated into multivariable diagnostic models, GDF-15 added significant value beyond established cardiac biomarkers. For prognosis, standalone biomarkers showed limited long-term discrimination, whereas multimarker approaches incorporating GDF-15 improved short-term mortality prediction and enhanced one-year risk stratification in patients with markedly elevated NT-proBNP levels. GDF-15 provides independent diagnostic and prognostic information in AHF and enhances multimarker strategies for comprehensive patient assessment, supporting its integration as a complementary biomarker in contemporary AHF evaluation.

## 1. Introduction

Acute heart failure (AHF) represents a complex clinical syndrome that requires urgent evaluation and treatment. It is defined either by a rapid de novo onset or by the acute worsening of symptoms and signs in patients with previously stable chronic heart failure (CHF). In clinical practice, hospital admissions occur in the context of acute decompensation, frequently triggered by precipitating factors such as myocardial ischemia, uncontrolled arterial hypertension (HTN), cardiac arrhythmias, pulmonary embolism, worsening renal function, systemic infections, or suboptimal adherence to chronic therapy. In contrast, cases of de novo AHF are encountered less frequently and are most commonly associated with acute coronary syndromes [1,2,3].

The clinical evaluation of patients with suspected AHF remains a significant challenge, as symptoms are frequently nonspecific and disease severity may be influenced by various comorbidities and interrelated pathophysiological mechanisms. Natriuretic peptides (NPs) and cardiac troponins (cTn) are widely used in clinical practice and provide valuable diagnostic and prognostic information in AHF, complementing clinical examination and imaging findings [4]. However, each biomarker reflects only certain aspects of the complex pathophysiology of AHF, which includes myocardial injury, congestion, inflammation, neurohormonal activation, endothelial dysfunction and systemic organ involvement. As a result, relying on a single biomarker frequently yields an incomplete clinical picture. Growing evidence supports the use of multimarker strategies that incorporate complementary biological pathways, with the potential to enhance diagnostic accuracy and risk stratification in AHF [5,6,7,8,9].

Recent studies have highlighted several emerging biomarkers that may provide additional information beyond traditional markers in patients with AHF. Among these are galectin-3, mid-regional pro-adrenomedullin, growth differentiation factor-15 (GDF-15), copeptin, soluble suppression of tumorigenicity 2 (sST2) and endothelin-1 (ET-1) [5,9,10,11,12,13,14,15].

GDF-15 has recently emerged as a biomarker of considerable interest in cardiovascular (CV) research, particularly in the context of HF. GDF-15 is a member of the Transforming Growth Factor beta (TGF-β) family and is expressed in numerous cell types, including cardiomyocytes, smooth muscle cells, fibroblasts, and endothelial cells, under the action of stressors [16,17,18]. Circulating levels of GDF-15 increase markedly in numerous pathologies, including CV, hepatic, renal diseases and cancer. Among CV conditions, higher GDF-15 concentrations have been identified in HTN, atrial fibrillation, peripheral artery disease, coronary syndromes and HF [17,19,20]. Research suggests that this cytokine may modulate cardiac remodeling and the adaptive response to myocardial stress [21].

Patients with HF present increased levels of GDF-15, which are directly correlated with disease severity and functional impairment and are independently associated with the risk of hospitalization and mortality [22,23,24]. In terms of diagnostic value, GDF-15 levels are significantly higher in patients with AHF compared to those with stable CHF, although its specificity for cardiac causes of dyspnea remains limited [25,26]. From a prognostic perspective, elevated GDF-15 concentrations have been associated with short- and long-term mortality and adverse outcomes, frequently offering independent predictive information [27,28,29].

The present study aimed to investigate the diagnostic value of GDF-15 in patients with AHF and to assess its performance beyond established cardiac biomarkers. We also explored the prognostic value of GDF-15 and its contribution within multimarker models for short- and long-term outcome prediction.

## 2. Materials and Methods

### 2.1. Study Design and Population

This study was designed as a prospective cohort study conducted in the Cardiology Clinic of the “St. Spiridon” Emergency County Hospital in Iași, Romania. Consecutive patients admitted with a primary diagnosis of AHF were prospectively enrolled at the time of hospitalization. Furthermore, the participants were followed for a period of 12 months to assess clinical outcomes, including short-term and long-term mortality.

A total of 102 participants were included in the final analysis, comprising 60 patients diagnosed with AHF and 42 control subjects. The diagnosis of AHF was established in accordance with current European Society of Cardiology (ESC) guidelines, based on typical symptoms and signs at presentation, elevated N-terminal pro–B-type natriuretic peptide (NT-proBNP) levels and echocardiographic evidence of cardiac dysfunction. The control group included individuals without a prior diagnosis of HF, as well as patients with stable, compensated CHF, without any recent HF-related hospitalization.

The exclusion criteria included age below 18 years, pregnancy, end-stage renal disease, terminal oncological disease or documented neuropsychiatric disorders. Patients presenting with active systemic inflammatory diseases or active malignancies at the time of admission were also excluded. Written informed consent was required for study participation.

The study was conducted in accordance with the ethical standards of the Declaration of Helsinki revised in 2013, and written informed consent was obtained from all participants, including those in the control group, prior to enrollment. The study protocol was reviewed and approved by the Ethics Committees of “Grigore T. Popa” University of Medicine and Pharmacy and the “St. Spiridon” Emergency Clinical Hospital in Iași.

### 2.2. Data Collection and Laboratory Assessment

All patients underwent a rigorous clinical evaluation, which included a detailed physical examination and medical history. Medical records and electronic hospital databases were used to obtain sociodemographic and clinical data. Vital signs, including systolic and diastolic blood pressure, heart rate, and peripheral oxygen saturation, were recorded at the time of admission to the Cardiology Clinic.

Blood samples for laboratory analysis were collected within the first 48 h of admission. The standard biochemical tests included renal function parameters, electrolytes, complete blood count, liver enzymes, lipid profile, inflammatory markers, and iron metabolism indices. Conventional cardiac biomarkers were measured in EDTA plasma samples as part of routine clinical protocols. Pathological cut-off values were defined as NT-proBNP > 300 pg/mL and high-sensitivity cardiac troponin I(hs-cTnI) > 29 ng/L, following current guideline recommendations. NT-proBNP and hs-cTnI were analyzed using an automated PATHFAST immunoanalyzer (PHC Corporation, Tokyo, Japan) based on chemiluminescent enzyme immunoassay methodology, according to routine laboratory practice and manufacturer instructions. Routine hematological and biochemical parameters were processed in the hospital central laboratory using standardized automated analyzers.

Plasma levels of GDF-15 were measured using an enzyme-linked immunosorbent assay kit (Human GDF-15 ELISA Kit, EIAAB Science, Wuhan, China, No. E2034h). Blood samples were obtained by venipuncture into potassium EDTA tubes and centrifuged within 30 min of collection at 1600× *g* for 15 min at 4 °C. The resulting plasma was aliquoted and stored at −80 °C until analysis. All samples were analyzed in a single batch to reduce inter-assay variability. The assay detection range was 15.6 to 1000 pg/mL, with a sensitivity below 7.8 pg/mL. Intra-assay and inter-assay coefficients of variation were below 5.3% and 8.2%, respectively, as indicated by the manufacturer. For samples with GDF-15 concentrations above the assay’s upper detection limit, serial dilutions were performed according to the manufacturer’s recommendations.

Echocardiographic examination was performed in all participants at admission using a General Electric Vivid^TM^ V7 ultrasound (GE Vingmed Ultrasound A/S, Horten, Norway). Left ventricular ejection fraction (LVEF) was measured using the biplane Simpson method. The structural evaluation included left ventricular (LV) end-diastolic diameter, interventricular septal thickness, and posterior wall thickness. We assessed right ventricular size and function through basal right ventricular diameter and tricuspid annular plane systolic excursion (TAPSE). We estimated pulmonary hemodynamics by measuring tricuspid regurgitation peak gradient (TRPG) and systolic pulmonary artery pressure (sPAP) when Doppler signals were clear. Other parameters included left and right atrial dilation, inferior vena cava (IVC) diameter, maximum aortic valve velocity, and the presence of aortic stenosis.

### 2.3. Statistical Analysis

Statistical analysis was performed using IBM SPSS Statistics for Windows, version 26. The normality of continuous variables was initially assessed using the Kolmogorov–Smirnov test, together with graphical inspection of histograms and Q-Q plots. Continuous variables are presented as mean ± standard deviation for descriptive purposes and as median with interquartile range for group comparisons when non-normal distributions were observed. Categorical variables were presented as absolute numbers and percentages.

Comparisons between patients with AHF and the control group were made using the independent samples *t*-test and the Mann–Whitney U test, depending on whether the variables were normally distributed or not. Categorical variables were compared using the chi-square test or Fisher’s exact test, as appropriate. Associations between circulating GDF-15 levels and clinical, laboratory, and echocardiographic parameters were evaluated using Spearman rank correlation analysis. Correlation coefficients and corresponding *p* values were reported.

The diagnostic performance of individual biomarkers for AHF was assessed using receiver operating characteristic (ROC) curve analysis. The area under the curve (AUC) with 95% confidence intervals was calculated to evaluate discriminative ability. To assess the independent diagnostic value of GDF-15, multivariable logistic regression models were constructed. Model 1 included age, GFR, NT-proBNP, and hs-cTnI. Model 2 additionally included GDF-15 to evaluate its incremental diagnostic contribution. Model performance was assessed using minus two log likelihood, Nagelkerke R-squared, the Hosmer–Lemeshow goodness-of-fit test, and overall classification accuracy.

For prognostic evaluation, receiver operating characteristic analyses were performed for in-hospital, 30-day, 3-month, 6-month, and 1-year mortality, with calculation of corresponding AUC values. Multivariable logistic regression models were constructed to assess 30-day mortality, including GDF-15, NT-proBNP, age, renal function, and LVEF. In addition, a stratified multimarker model that included GDF-15 was developed for 1-year mortality according to the NT-proBNP median value, based on its role as the reference biomarker in AHF. Model performance was tested independently for each subgroup.

All statistical tests were two-sided, and a *p*-value below 0.05 was considered statistically significant.

## 3. Results

The results are presented as baseline characteristics of the study population, followed by correlation analyses between GDF-15 and relevant clinical, laboratory, and echocardiographic parameters, as well as diagnostic performance, prognostic evaluation across follow-up intervals, and multimarker analyses.

### 3.1. Baseline Characteristics

The study cohort consisted of 102 patients, 60 of whom had AHF and 42 served as controls. The two groups were similar in terms of age, gender distribution, body mass index, blood pressure, smoking status, length of hospital stay, and hospitalization expenditures, with no statistically significant differences identified. On the other hand, AHF patients had higher heart rates and significantly decreased peripheral oxygen saturation at admission. As expected, patients in the AHF group showed more signs of volume overload and congestion, such as peripheral edema, pleural effusion, and pulmonary rales, compared to controls (*p* < 0.001). In addition, they were less likely to have sinus rhythm (Table 1).

The presence of the most frequent comorbid conditions known to contribute to HF progression was investigated in patients with AHF. Overall, a higher burden of cardiovascular and metabolic comorbidities was observed in this group compared with controls, consistent with a more complex clinical presentation at admission. Differences between groups were particularly evident for ischemic heart disease, diabetes mellitus, chronic kidney disease, and anemia, while HTN and dyslipidemia were common in both populations.

The baseline laboratory parameters at admission are described in Supplementary Table S1. Patients with AHF had significantly higher circulating levels of NT-pro-BNP and GDF-15 compared with controls (*p* < 0.01). Markers of myocardial injury and cellular damage, including hs-cTnI and LDH, were also higher in the AHF group. Inflammatory activity varied significantly between groups, with patients suffering from AHF having greater CRP levels and leukocyte counts. In addition, renal function parameters demonstrated significant impairment in the AHF group, with increased urea and creatinine concentrations (*p* < 0.001) and lower estimated glomerular filtration rate values (*p* = 0.005). Hepatic biomarkers also varied significantly between groups, with higher AST, ALT and GGT observed among patients with AHF. Other notable differences were observed in metabolic and iron-related parameters. There were no significant differences for CK, CK-MB, triglycerides, ferritin, TSH, or electrolytes. In addition to detailed clinical examination and laboratory assessment, all patients underwent comprehensive echocardiographic evaluation. Significant structural and functional differences were found between individuals with AHF and the control group (Table 2). Atrial remodeling was considerably more prevalent in the AHF group, with significantly higher rates of both left and right atrial dilation compared with controls (*p* < 0.001). Patients with AHF also had larger ventricular dimensions and significantly lower LVEF than controls (*p* < 0.001), indicating decreased systolic function. Additionally, there were notable differences in loading conditions and right ventricular function markers between the groups. Patients with AHF showed an increased IVC diameter, raised sPAP, and higher right-sided pressure gradients, all indicating elevated filling pressures and probable pulmonary hypertension. There were no statistically significant differences between groups in terms of interventricular septal thickness, posterior wall thickness of the left ventricle, maximum aortic flow velocity, or aortic stenosis. Overall, the echocardiographic profile of patients with AHF was defined by biventricular dysfunction, atrial enlargement, and higher filling pressures.

In the AHF cohort, all three biomarkers demonstrated markedly elevated concentrations compared to the control group. Serum GDF-15 levels were significantly higher in patients with AHF (median 655.9 pg/mL, IQR 465.1) compared to controls (282.5 pg/mL, IQR 161.2) (Z = −6.412, *p* < 0.001). The AHF group also had significantly higher levels of NT-proBNP and hs-cTnI (*p* < 0.001 for both) (Table 3).

### 3.2. GDF-15 and Relevant Clinical, Laboratory and Echocardiographic Parameters

To characterize the clinical relevance of GDF-15, we evaluated the relationships between circulating GDF-15 levels and clinical, laboratory, and echocardiographic parameters (Supplementary Table S2). GDF-15 demonstrated a significant positive correlation with age (r = 0.32, *p* = 0.01) and NT-proBNP levels (r = 0.32, *p* = 0.01). Among echocardiographic parameters, higher GDF-15 levels were observed in relation to larger LV end-diastolic diameter (r = −0.25, *p* = 0.04) and reduced right ventricular systolic function, as reflected by TAPSE (r = −0.35, *p* < 0.01), but not with LVEF. No significant associations were identified between GDF-15 and vital signs, including systolic and diastolic blood pressure, heart rate, and SaO2.

**Figure 1:**
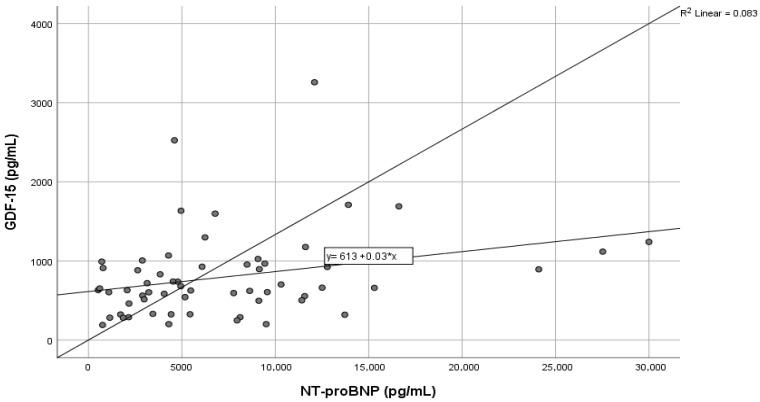
Correlation between GDF-15 and NT-proBNP levels in AHF patients.

In addition, several laboratory markers exhibited significant relationships with GDF-15 in AHF patients. Positive associations were observed with renal dysfunction indicators such as creatinine (r = 0.38, *p* < 0.01) and urea (r = 0.30, *p* = 0.01), along with a strong inverse correlation with GFR (r = −0.51, *p* < 0.01). Other important associations were seen with ET-1 (r = 0.39, *p* < 0.01), LDH (r = 0.36, *p* < 0.01), potassium (r = 0.34, *p* < 0.01), and alkaline reserve (r = −0.30, *p* = 0.01). Total cholesterol, hemoglobin and serum iron levels showed an inverse relationship with GDF-15. Given that NT-proBNP is the standard biomarker for the evaluation of AHF, a correlation analysis was performed to investigate the relationship between the new biomarker GDF-15 and NT-proBNP levels. Figure 1 shows the corresponding scatter plot. GDF-15 and NT-proBNP levels had a small but statistically significant positive correlation, according to the analysis (r = 0.287, *p* = 0.026). This indicates that patients with higher NT-proBNP levels, which are indicative of more severe HF and fluid overload, also tended to have slightly higher GDF-15 levels.

**Figure 2:**
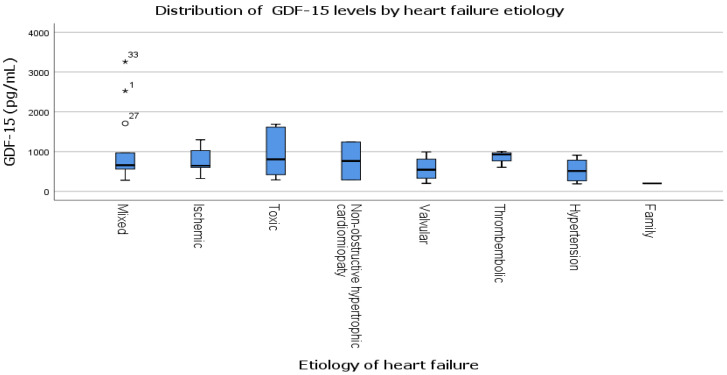
Distribution of GDF-15 by HF etiology. Boxplots show median, interquartile range, and range. Circles (°) represent outliers, asterisks (*) represent extreme outliers, with numbers indicating individual case identifiers.

Regarding the concentrations of GDF-15 across different etiologies of AHF (Figure 2), we observed notable variability, with the highest median values found in the toxic and non-obstructive hypertrophic cardiomyopathy subgroups. However, the disparities in mean GDF-15 values among the etiological categories did not reach statistical significance. Even with several outliers in the toxic and mixed etiology subgroups, there was no clear pattern that showed a strong link between the cause of HF and the level of GDF-15.

**Figure 3:**
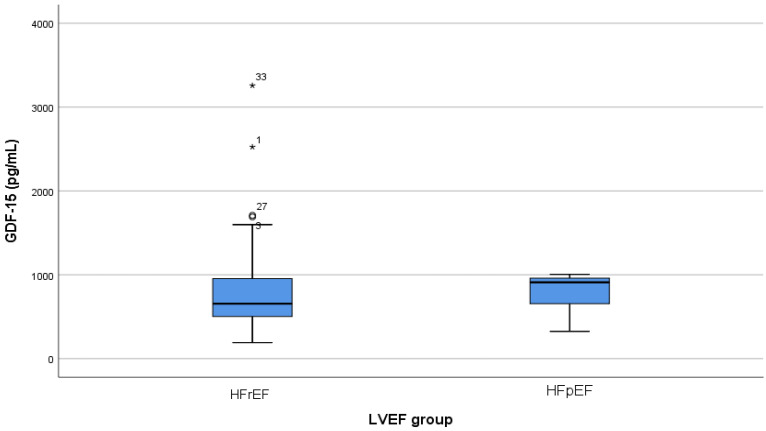
Distribution of GDF-15 by LVEF. Boxplots show median, interquartile range, and range. Circles (°) indicate outliers, asterisks (*) indicate extreme outliers, with numbers representing individual case identifiers.

Given the inconsistent results in the literature regarding the relationship between GDF-15 and LVEF, the link between circulating GDF-15 levels and LVEF was further investigated in our study cohort. An analysis of GDF-15 levels based on LVEF demonstrated a trend toward higher concentrations in patients with decreased LVEF (HFrEF) compared to those with preserved LVEF (HFpEF) (Figure 3). However, the difference between the two groups was not statistically significant (*p* = 0.862).

### 3.3. Diagnostic Role of GDF-15 in AHF

**Figure 4:**
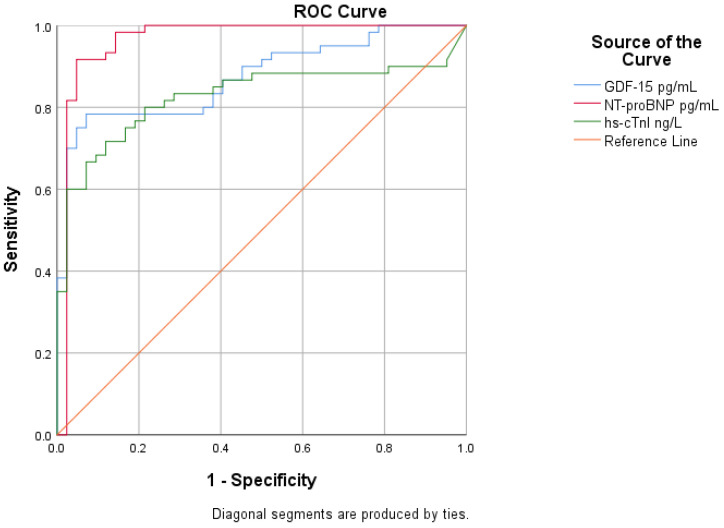
ROC curves for GDF-15, NT-proBNP and hs-cTnI for the diagnosis of AHF.

ROC curve analysis was performed to assess the diagnostic performance of GDF-15 in patients with AHF and to compare it with established cardiac biomarkers. GDF-15 showed good diagnostic performance for AHF, with an AUC of 0.874 (*p* < 0.001). NT-proBNP showed the highest diagnostic accuracy, with an AUC of 0.963 (*p* < 0.001), confirming its essential role in the diagnosis of AHF. Hs-cTnI demonstrated a lower diagnostic performance, with an AUC of 0.827, but it was still significant (*p* < 0.001). As a result, GDF-15 demonstrated diagnostic accuracy superior to hs-cTnI but inferior to NT-proBNP, suggesting a meaningful but complementary role in the diagnostic evaluation of AHF (Figure 4, Table 4).

Based on the coordinates of the ROC curve, a GDF-15 concentration of approximately 455 pg/mL was identified as the most appropriate threshold for diagnostic discrimination. At this cut-off value, GDF-15 achieved a sensitivity of 78.3% and a specificity of 92.9%.

In order to evaluate the independent diagnostic value of GDF-15 in AHF, two multivariable logistic regression models were constructed. The baseline model (Model 1) included traditional factors such as age, renal function assessed by GFR, NT-proBNP, and hs-cTnI. In this model, none of the conventional biomarkers reached independent statistical significance, even though the overall model demonstrated good fit and calibration. The model explained 53.6% of the variance in diagnosis and correctly classified 79.0% of cases, with excellent calibration according to the Hosmer–Lemeshow test (*p* = 0.997).

Following the addition of GDF-15, the extended model (Model 2) showed a significant improvement in diagnostic performance. The inclusion of GDF-15 resulted in a marked reduction in minus 2 log likelihood (Δ minus 2LL = 22.02, *p* < 0.001) and a substantial increase in explained variance (Nagelkerke R^2^ increased from 0.536 to 0.758). Model calibration remained excellent (Hosmer–Lemeshow *p* = 0.94), and overall classification accuracy increased to 91.4% (Table 5).

In the fully adjusted model, GDF-15 emerged as an independent diagnostic predictor of AHF (B = 0.008, *p* = 0.006, OR = 1.008, 95% CI 1.002–1.014), while hs-cTnI also retained independent significance (B = 0.141, *p* = 0.042, OR = 1.151, 95% CI 1.005–1.319). In contrast, NT-proBNP, age, and renal function did not show independent significance after adjustment. The full multivariable results are presented in Table 6.

### 3.4. Prognostic Value of GDF-15 in AHF

**Figure 5:**
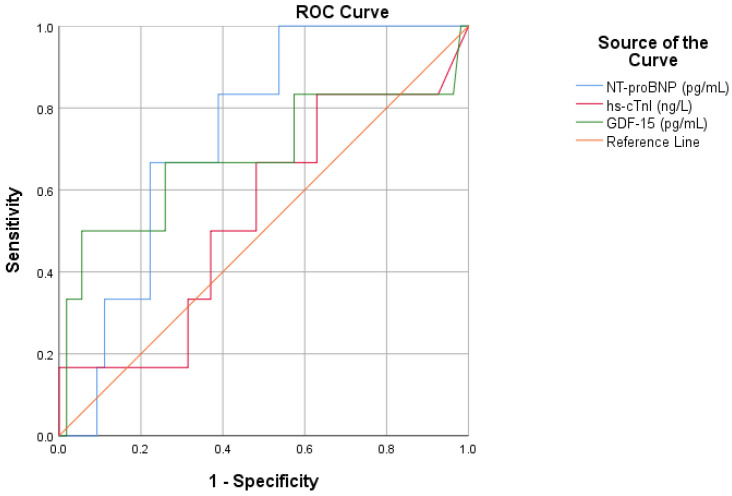
ROC curve for the relationship between cardiac biomarkers and in-hospital mortality rates.

To evaluate each biomarker’s ability to predict in-hospital mortality, we created receiver operating ROC curves. With an area AUC of 0.738 (*p* = 0.058), NT-proBNP demonstrated the greatest capacity for discrimination among the three biomarkers, almost reaching statistical significance. GDF-15 had an AUC of 0.684 (*p* = 0.143), indicating a modest level of predictive power. The discriminatory power of hs-cTnI, on the other hand, was limited, with an AUC of 0.540 (*p* = 0.749) (Figure 5, Table 7).

**Figure 6:**
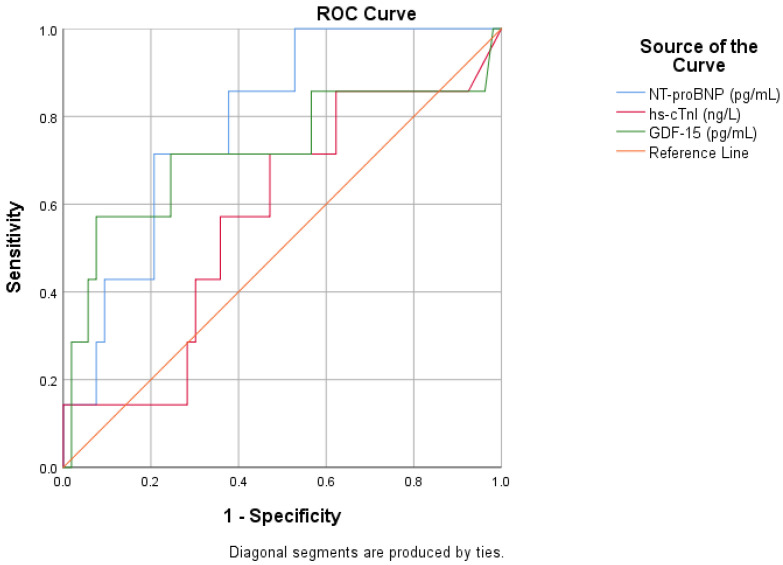
ROC curve for the relationship between cardiac biomarkers and 30-day mortality rate.

In the prediction of 30-day mortality, NT-proBNP showed the highest discriminatory performance, with an AUC of 0.787 and a significant prognostic value. GDF-15 demonstrated moderate predictive ability, with an AUC of 0.721, approaching statistical significance, whereas hs-cTnI had limited predictive value. The optimal cutoff for GDF-15 in this cohort was 620 pg/mL (Figure 6, Table 8).

At intermediate and long-term follow-up, the discriminatory performance of individual biomarkers gradually declined. At three months, GDF-15 showed the highest AUC among the evaluated biomarkers, followed by NT-proBNP, whereas hs-cTnI demonstrated the lowest discriminatory capacity. However, none of the biomarkers reached statistical significance at this time point. A similar pattern was observed at 6 months and one year, with GDF-15 consistently showing numerically higher AUC values compared with NT-proBNP and hs-cTnI, although without statistical significance. Detailed ROC analyses for these follow-up intervals are provided in Supplementary Figures S1–S3 and Supplementary Tables S3–S5.

### 3.5. Multimarker Strategy for Prognostic Assessment in AHF

Given the modest prognostic performance of individual biomarkers across follow-up intervals, GDF-15 showed a relatively stronger discriminatory ability for short-term mortality. Based on this observation, we explored a multimarker approach combining GDF-15, NT-proBNP and hs-cTnI to integrate complementary pathophysiological pathways. A composite score was defined as the number of biomarkers exceeding predefined cutoffs: NT-proBNP greater than 5600 pg/mL, hs-cTnI greater than 50 ng/L, and GDF-15 greater than 850 pg/mL.

**Figure 7:**
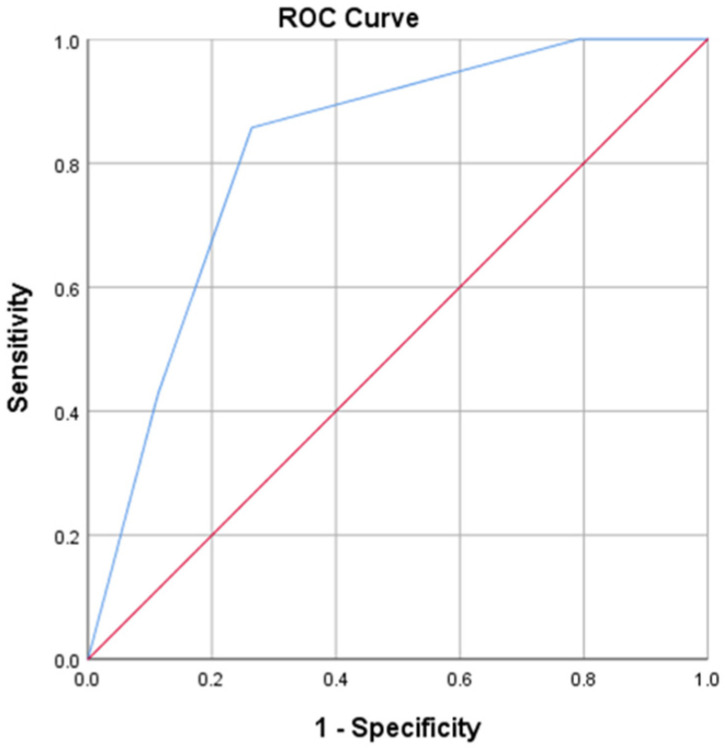
ROC curve of the multimarker model for prediction of 30-day mortality in AHF.

The multimarker model demonstrated good prognostic performance for 30-day mortality, with an AUC of 0.819 (*p* < 0.01). Increasing values were associated with a stepwise increase in mortality risk. The optimal cut-off of the multimarker model was ≥2 positive biomarkers, with sensitivity = 85.7% and specificity = 75.0% for 30-day mortality, outperforming each biomarker individually (Figure 7, Table 9).

Given the limited prognostic accuracy of individual biomarkers for 1-year mortality, a multimarker logistic regression model was constructed to evaluate whether the combination of GDF-15 with established clinical and biochemical predictors improves risk stratification. NT-proBNP was used for patient stratification based on its role as the reference biomarker for HF, and patients were subsequently analyzed according to the NT-proBNP median value of 5304 pg/mL. Model performance was evaluated separately for each category.

In patients with NT-proBNP ≥ 5304 pg/mL, the multimarker model was statistically significant (χ^2^ = 12.718, *p* = 0.026), explaining 47.2% of the variance in one-year mortality (Nagelkerke R^2^ = 0.472), with excellent calibration (Hosmer–Lemeshow *p* = 0.807). In contrast, the model was not significant in patients with NT-proBNP < 5304 pg/mL (χ^2^ = 7.299, *p* = 0.199), with a lower explained variance (Nagelkerke R^2^ = 0.306). Table 10 provides a summary of all model performance metrics.

## 4. Discussion

AHF represents a critical stage in the progression of cardiac conditions and is associated with a high risk of complications and mortality. The heterogeneous clinical presentation of this condition complicates early diagnostic and prognostic assessment. As a result, the accurate evaluation of patients with AHF remains challenging, as clinical presentation and disease severity are influenced by both cardiac dysfunction and systemic involvement. Moreover, the presence of multiple comorbidities and overlapping pathophysiological processes may limit the ability of clinical assessment alone to reliably estimate risk and guide early management decisions [1,30].

In this clinical context, circulating biomarkers have become an important component of patient evaluation, complementing clinical examination and imaging findings. cTn and NPs are widely used in clinical practice, reflecting myocardial injury and hemodynamic stress, respectively. However, while NPs are highly sensitive, their specificity is limited and intermediate values define a diagnostic gray zone in which clinical interpretation becomes more uncertain. Complementary evaluation of other biomarkers is crucial in this context, especially those that represent different pathophysiological processes related to HF [31,32].

Among the novel biomarkers extensively investigated in the literature, GDF-15 represents an appealing candidate for evaluation in AHF, as it reflects biological processes that extend beyond isolated myocardial stress. This may be particularly relevant in the diagnostic gray zone of NPs, where additional biomarkers can help clarify complex clinical presentations. Most of the existing evidence on GDF-15 comes from research involving patients with CHF, and data in acute situations are still limited. However, several studies have already indicated that GDF-15 could have diagnostic and prognostic value in AHF, supporting further evaluation of GDF-15 in this setting [33].

In this study, patients with AHF differed significantly from the control group in clinical, laboratory, and echocardiographic parameters, reflecting more severe congestion and systemic involvement. These findings are consistent with previous results linking elevated GDF-15 levels to disease severity and functional impairment in HF [34,35]. The two groups were comparable in terms of age and sex distribution, with no statistically significant differences. In addition, patients with AHF tended to have longer hospital stays and higher hospitalization costs compared with controls, highlighting the socioeconomic impact of this disease.

Biochemical analysis revealed significantly higher levels of NT-proBNP, hs-cTnI, and GDF-15 in patients with AHF. Among these biomarkers, GDF-15 demonstrated the greatest relative increase, suggesting a potential role as an additional marker in the acute setting. Patients with AHF exhibited impaired renal function, increased inflammatory and hepatic markers, along with lower hemoglobin and serum iron levels than controls. Additional structural and functional changes were revealed by echocardiographic evaluation, with the AHF group presenting more frequent atrial dilation, larger ventricular dimensions, and reduced LVEF and TAPSE.

Consistent with previous findings, GDF-15 levels in our study showed a positive correlation with age. This observation aligns with epidemiological data indicating that the prevalence of CHF with acute decompensation increases markedly with advancing age, exceeding 10% among individuals older than 70 years in developed countries [34].

Transthoracic echocardiography is considered the most useful imaging tool in establishing the etiology of HF and guiding therapeutic decisions. In our analysis, GDF-15 had a negative correlation with LVEF, although its concentrations were not significantly impacted by LVEF (*p* = 0.67, r = −0.05). Similar findings were reported by Miftode et al. However, prior studies have reported conflicting findings regarding the association between GDF-15 and LVEF. Given these inconsistencies, we further examined this relationship in our cohort. GDF-15 levels tended to be higher among patients with HFrEF compared with those with HFpEF, although this difference did not reach statistical significance (Figure 3, *p* = 0.862). This observation is clinically relevant, as 30 to 50% of patients hospitalized for AHF present with pEF, highlighting the potential utility of GDF-15 across the full spectrum of HF phenotypes [14,32]. Chan et al. found that combining NT-proBNP and GDF-15 improves diagnostic accuracy in distinguishing between HFpEF and HFrEF compared to using each marker separately [36].

Taken together, these data indicate that GDF-15 reflects a wider biological response to acute cardiac decompensation rather than being a marker only associated with LV systolic performance. The favorable correlation with age is most likely due to the cumulative burden of chronic cardiovascular stress, systemic inflammation, and comorbidities, all of which increase with age. In addition, the association between GDF-15 and NT-proBNP suggests that both biomarkers rise in response to hemodynamic stress, despite the fact that they represent somewhat different pathophysiological mechanisms. The absence of a significant relationship between GDF-15 and LVEF further supports the concept that GDF-15 captures global myocardial and systemic stress beyond isolated contractile impairment, which may explain its elevation across the full spectrum of HF phenotypes, including patients with HFpEF.

Endothelial dysfunction is one of the first stages in the development of CV diseases. In patients with CHF, the presence of endothelial damage is associated with increased mortality. Numerous studies suggest that GDF-15 influences endothelial function by altering vascular contraction and relaxation mechanisms, which may promote both macrovascular and microvascular dysfunction, both of which contribute to progressive deterioration of cardiac function. On the other hand, GDF-15 can stimulate endothelial cell proliferation during angiogenesis, but under certain conditions it also contributes to endothelial senescence. ET-1 is a recognized marker of endothelial dysfunction and the most effective endogenous vasoconstrictor discovered to date [13,14,21,37,38,39]. In our investigation, circulating GDF-15 levels were strongly and significantly associated with ET-1, indicating a direct relationship between the new biomarker and endothelial activation in patients with AHF.

In the setting of AHF, the association between GDF-15 and renal function holds particular interest. Our results revealed significant positive correlations between GDF-15 and urea and creatinine, as well as a strong inverse correlation with GFR, suggesting an important interaction between cardiac dysfunction and renal impairment in acute decompensation. Previous studies have shown that GDF-15 may increase in response to inflammatory, oxidative, and ischemic stress, with circulating levels rising in parallel with declining renal function and increased CV risk [40,41]

In line with previous research, GDF-15 has been linked to anemia and iron deficiency, both of which are common in HF and associated with poor clinical outcomes. The negative correlations between GDF-15, hemoglobin, and serum iron in our AHF cohort support these findings, implying that GDF-15 may capture the contribution of anemia and iron deficiency, which can act not only as disease severity markers but also as potential precipitating factors for acute decompensation [42].

Elevated GDF-15 levels have been observed in experimental models of cardiac remodeling, pressure overload, and cardiomyopathy, suggesting activation in response to myocardial stress [43]. In line with these observations, our clinical data demonstrated a positive correlation between circulating GDF-15 and NT-proBNP levels in patients with AHF (Figure 1). This finding indicates that both biomarkers reflect similar pathways of cardiac overload and injury, though they are not identical. However, while NPs primarily reflect hemodynamic stress, GDF-15 may also capture other systemic and cellular stress signals. The moderate strength of this correlation supports the idea that GDF-15 provides additional information and reinforces its potential role in a multimarker approach in acute situations.

Although GDF-15 is known to be elevated in various CV pathologies, our analysis demonstrated heterogeneous GDF-15 distributions across different etiologies of AHF, with higher median values and wider dispersion observed in mixed, ischemic, and toxic etiologies (Figure 2).

In recent years, GDF-15 has become an essential biomarker in HF due to its involvement in the incidence, diagnosis, progression, and prognosis of the disease. Elevated GDF-15 levels have been consistently associated with an increased risk of developing HF, including forms occurring after myocardial infarction [33,44,45]. In our study, GDF-15 showed robust diagnostic performance and provided additional information beyond traditional cardiac biomarkers. The identified cut-off value was associated with high specificity and acceptable sensitivity, supporting the potential role of GDF-15 in multimarker diagnostic strategies. Multivariable analysis further emphasized the diagnostic value of GDF-15. When included in a model incorporating age, renal function, NT-proBNP, and hs-cTnI, the model’s performance and overall diagnostic accuracy improved significantly. Notably, in the fully adjusted model, GDF-15 stood out as an independent diagnostic predictor of AHF. Overall, these findings support the role of GDF-15 as an important part of multimarker diagnostic approaches in AHF, complementing established biomarkers and enhancing diagnostic performance.

AHF is associated with high mortality both during hospitalization and after discharge. Numerous studies have proven GDF-15’s substantial prognostic importance in HF, since this biomarker is strongly associated with mortality and adverse disease progression. For example, Lok et al. and Kempf et al. found that GDF-15 is an independent predictor of mortality in CHF [27,34]. In the present study, GDF-15 showed a consistent prognostic profile across short-term follow-up. For in-hospital and 30-day mortality, GDF-15 demonstrated moderate discriminative ability, comparable to NT-proBNP and superior to hs-cTnI. Although statistical significance was not reached at all time points, the numerical performance of GDF-15 remained stable and clinically relevant. However, the prognostic significance of GDF-15 in AHF has been explored in a relatively small number of studies, both for short-term and long-term outcomes.

In our study, GDF-15 demonstrated a consistent prognostic profile across short-term and long-term follow-up. For in-hospital and 30-day mortality, GDF-15 showed moderate ability to distinguish outcomes, similar to NT-proBNP, but superior to hs-cTnI. While statistical significance was not achieved at all time points, the numerical performance of GDF-15 remained stable and clinically relevant. These results are concordant with previous reports indicating that elevated admission levels of GDF-15 are independently associated with early mortality in AHF, even after adjustment for NT-proBNP and cTn. Beyond admission values, previous studies have emphasized the importance of dynamic changes in GDF-15 during hospitalization. A decrease in GDF-15 levels from admission to discharge has been associated with a reduced risk of rehospitalization and improved short-term outcomes [28]. Although serial measurements were not available in our study, the observed prognostic performance of GDF-15 supports its potential role in early risk stratification.

At intermediate and long-term follow-up, individual biomarkers showed limited prognostic accuracy. This finding may be partly explained by the relatively small size of the AHF cohort and the limited number of long-term outcome events, which reduced the statistical power to detect independent prognostic effects. In addition, heterogeneity in clinical characteristics and post-discharge management may have further influenced long-term outcomes. Nevertheless, GDF-15 consistently showed numerically higher AUC values than NT-proBNP and hs-cTnI at three months, six months, and one year (detailed ROC analyses are provided in the Supplementary Materials). This observation is consistent with previous studies and meta-analyses identifying GDF-15 as a strong predictor of all-cause mortality in HF and supports its potential role in multimarker prognostic strategies. Interestingly, some studies synthesized by George et al. suggest that the prognostic value of GDF-15 may even surpass that of NT-proBNP in certain groups [29].

The value of GDF-15 becomes particularly evident within multimarker approaches. The modest prognostic performance of individual biomarkers at intermediate and longer follow-up highlights that relying on a single biomarker may not be sufficient for risk assessment in acute heart failure. In our study, combining GDF-15 with NT-proBNP and hs-cTnI significantly improved short-term mortality prediction at 30 days, achieving good overall discriminative performance and surpassing each biomarker individually. These results support earlier evidence that multimarker panels incorporating GDF-15 offer better prognostic accuracy than single biomarker methods [46,47].

For long-term prognosis, the multimarker logistic regression model demonstrated significant predictive value for one-year mortality in patients with elevated NT-proBNP levels. In this higher-risk subgroup, GDF-15 contributed substantially to the explained variance, likely reflecting improved risk discrimination in patients with more advanced disease severity. Similar observations were reported in the PARADIGM-HF trial, where baseline GDF-15 levels and their longitudinal changes were independently associated with mortality and cardiovascular events. In this higher-risk subgroup, GDF-15 was one of the most important variables and significantly contributed to the explained variance. This observation likely reflects enhanced risk discrimination in patients with advanced disease severity and higher event rates, rather than suggesting that GDF-15 is useful only in critically ill patients. This finding coincides with results from the PARADIGM HF trial, where baseline GDF-15 levels and ongoing increases were independently linked to mortality, CV events, and hospitalizations, regardless of the treatment strategy [48]. Comparable findings were also described by Hao et al., who reported improved prognostic accuracy when GDF-15 was combined with NT-proBNP [46]. Taken together, these findings strengthen the idea that GDF-15 provides independent and additional prognostic information in AHF.

**Figure 8:**
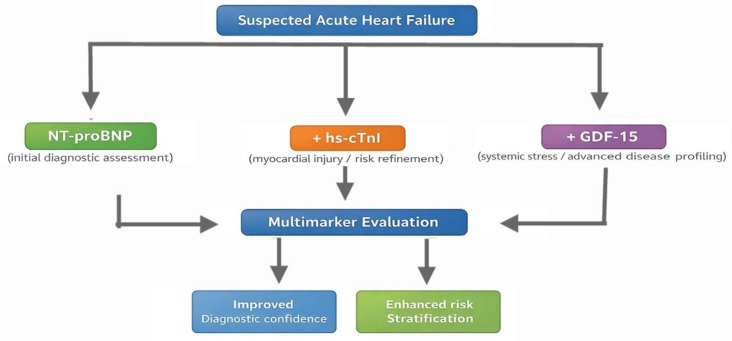
Multimarker framework for the evaluation of suspected AHF.

Overall, these findings support the role of GDF-15 as a promising component of multimarker strategies in AHF, providing complementary diagnostic and prognostic information and improving risk stratification when integrated with established cardiac biomarkers. Figure 8 illustrates the conceptual integration of these biomarkers within a multimarker framework.

## 5. Limitations of the Study

Several limitations should be considered when interpreting the results. Firstly, this was a single-center study with a relatively modest sample size, which may limit the generalizability of the findings. However, the prospective design and the consistent direction of the observed associations support the internal validity of the results. In addition, the relatively small and heterogeneous control group limited the feasibility of separate subgroup analyses. Secondly, studying dynamic changes in GDF-15 levels during hospitalization and after discharge follow-up was not possible since biomarker measures were taken at a single time point after admission. Finally, external validation in larger, multicenter cohorts is necessary before broad clinical implementation of the proposed multimarker strategies.

## 6. Conclusions

The present study highlights the clinical relevance of GDF-15 as an integrative biomarker in AHF. GDF-15 demonstrated good discriminative ability for the diagnosis of AHF and added significant value beyond established cardiac biomarkers when incorporated into multivariable diagnostic models. In addition to its diagnostic contribution, GDF-15 provided relevant prognostic information, particularly when integrated into multimarker approaches. Its inclusion improved short-term mortality prediction and enhanced long-term risk stratification in patient subgroups characterized by higher disease severity, such as those with markedly elevated NT-proBNP levels. These findings support the integration of GDF-15 into comprehensive multimarker strategies for AHF, with the potential to improve both diagnostic accuracy and prognostic evaluation in acute clinical settings. Larger multicenter studies are necessary to further substantiate these results and to establish the optimal clinical application of GDF-15 in AHF assessment.

## Figures and Tables

**Table 1:** Baseline characteristics.

Variable	Total (n = 102)	AHF Group (n = 60)	Control Group (n = 42)	*p*-Value
Age (mean ± SD)	67.96 ± 13.2	68.4 ± 13.14	67.33 ± 13.43	0.690
Gender				0.304
Female n (%)	33 (32.4%)	17 (28.3%)	16 (38.1%)	
Male n (%)	69 (67.6%)	43 (71.7%)	26 (61.9%)	
Length of stay, days (mean ± SD)	7.94 ± 13.2	8.65 ± 7.22	6.93 ± 5.33	0.192
Hospitalization costs, lei (mean ± SD)	15,433.34 ± 16,902.24	17,653 ± 20,271.75	12,262.4 ± 9741.36	0.113
BMI, kg/m^2^	27.92 ± 5.63	28.2 ± 6.12	27.12 ± 3.93	0.454
SBP, mmHg	130.29 ± 20.24	129.42 ± 21.61	131.55 ± 18.29	0.603
DBP, mmHg	80.63 ± 12.62	80.45 ± 13.3	80.88 ± 11.72	0.866
Heart rate, bpm	86.40 ± 26.7	91.52 ± 27.59	79.1 ± 23.95	0.02
SpO2, %	93.91 ± 4.09	92.08 ± 4.19	96.52 ± 2.02	<0.001
Active smoker	20 (19.6%)	9 (15%)	11 (26.2%)	0.164
Alcohol abuse	46 (45.1%)	30 (50%)	16 (38.1%)	0.239
Peripheral edema	48 (47.5%)	39 (66.1%)	9 (21.4%)	<0.001
Pleural effusion	31 (30.4%)	29 (48.3%)	2 (4.8%)	<0.001
Pulmonary rales	44 (43.1%)	43 (71.7%)	1 (2.4%)	<0.001
Sinus rhythm	61 (59.8%)	28 (46.7%)	33 (78.6%)	0.001

Legend: n—number; SD—standard deviation; SpO2—oxygen saturation; AHF—acute heart failure; BMI—body mass index; SBP—systolic blood pressure; DBP—diastolic blood pressure.

**Table 2:** Baseline echocardiographic parameters.

Parameter	Total (n = 102)	AHF Group (n = 60)	Control Group (n = 42)	*p*-Value
Left atrium dilated (N, %)	65 (63.7%)	49 (81.7%)	16 (38.1%)	<0.001
Right atrium dilated (N, %)	62 (60.8%)	50 (83.3%)	12 (28.6%)	<0.001
Right ventricle diameter (mm)	34.36 ± 6.31	36.06 ± 7.14	31.95 ± 3.81	<0.001
LV end-diastolic diameter (mm)	52.29 ± 8.96	54.35 ± 9.85	49.36 ± 6.55	0.003
Interventricular septum (mm)	11.73 ± 2.05	12.03 ± 2.35	11.29 ± 1.47	0.051
Posterior wall of LV (mm)	10.98 ± 1.61	11.12 ± 1.86	10.79 ± 1.15	0.272
Maximum velocity in the aorta (m/s)	1.57 ± 0.71	1.59 ± 0.86	1.55 ± 0.43	0.799
Aortic stenosis (N, %)	9 (8.8%)	7 (11.7%)	2 (4.8%)	0.199
LVEF (%)	38.43 ± 17.55	29.05 ± 15.06	51.83 ± 10.94	<0.001
IVC (mm)	17.50 ± 4.79	19.38 ± 4.6	14.81 ± 3.67	<0.001
sPAP (mmHg)	36.73 ± 13.6	42.07 ± 13.24	27.02 ± 7.64	<0.001
TRPG (mmHg)	28.73 ± 13.05	33.28 ± 13.45	21.14 ± 7.92	<0.001
TAPSE (mm)	19.08 ± 3.8	18.13 ± 3.78	20.90 ± 3.16	0.001

Legend: n—number of patients, N—number, AHF—acute heart failure, LV—left ventricle, LVEF—left ventricular ejection fraction, TAPSE—tricuspid annular plane systolic excursion, sPAP—systolic pulmonary artery pressure, TRPG—tricuspid regurgitation pressure gradient, IVC—inferior vena cava.

**Table 3:** Comparison of cardiac biomarker levels between patients with AHF and control group.

Biomarker	ICA (n = 60) Median (IQR)	Control Group (n = 42) Median (IQR)	Mean Rank ICA	Mean Rank Control Group	Z	*p*-Value
GDF-15 (pg/mL)	655.9 (465.1)	282.1 (161.2)	67.22	29.05	6.412	<0.001
NT-proBNP (pg/mL)	5304 (6640.5)	183.5 (363)	70.95	23.71	7.935	<0.001
hs-cTnI (ng/L)	50.1 (267.7)	3.6 (6.4)	65.23	31.88	5.604	<0.001

**Table 4:** Detailed analysis of AUC: diagnostic performance of GDF-15 compared to NT-proBNP and hs-cTnI.

Test Result Variable(s)	Area	Std. Error	*p*-Value	95% Confidence Interval	
				Lower Bound	Upper Bound
GDF-15 pg/mL	0.874	0.034	<0.001	0.807	0.941
NT-proBNP pg/mL	0.963	0.024	<0.001	0.915	1.000
Hs-cTnI ng/L	0.827	0.043	<0.001	0.743	0.911

**Table 5:** Comparative diagnostic model performance with and without GDF-15 in AHF.

Model	Covariates	−2 Log Likelihood	Nagelkerke R^2^	Δ−2LL vs. Previous	ΔR^2^	Hosmer–Lemeshow *p*	Overall Accuracy (%)
Model 1	Age, GFR, NT-proBNP, hs-cTnI	55.87	0.536	—	—	0.997	79.0
Model 2	Model 1 + GDF-15	33.85	0.758	22.02	0.222	0.941	91.4

**Table 6:** Predictive variables in the final multivariate logistic regression model (Model 2, including GDF-15).

	B	S.E.	Wald	df	*p*-Value	Exp(B)	95% C.I. for Exp(B)	
							Lower	Upper
Age	0.085	0.055	2.354	1	0.125	1.089	0.977	1.214
NT-proBNP pg/mL	0.000	0.000	1.475	1	0.225	1.000	1.000	1.000
GFR mL/min/1.73 m^2^	0.023	0.019	1.504	1	0.220	1.024	0.986	1.063
Hs-cTnI ng/L	0.141	0.069	4.126	1	0.042	1.151	1.005	1.319
GDF-15 pg/mL	0.008	0.003	7.582	1	0.006	1.008	1.002	1.014
Constant	−12.436	5.638	4.865	1	0.027	0.000		

**Table 7:** Detailed analysis of the AUC: GDF-15, NT-proBNP, and hs-cTnI correlation to in-hospital mortality rates.

Test Result Variable	Area	Std. Error	*p*-Value	95% Confidence Interval	
				Lower Bound	Upper Bound
NT-proBNP (pg/mL)	0.738	0.079	0.058	0.583	0.893
hs-cTnI (ng/L)	0.540	0.128	0.749	0.290	0.791
GDF-15 (pg/mL)	0.684	0.147	0.143	0.396	0.971

**Table 8:** Detailed analysis of the AUC: cardiac biomarkers and 30-day mortality rate.

Test Result Variable	Area	Std. Error	*p*-Value	95% Confidence Interval	
				Lower Bound	Upper Bound
NT-proBNP (pg/mL)	0.787	0.076	0.014	0.639	0.936
hs-cTnI (ng/L)	0.571	0.115	0.542	0.346	0.796
GDF-15 (pg/mL)	0.721	0.130	0.059	0.466	0.976

**Table 9:** Diagnostic performance of the multimarker model for prediction of 30-day mortality in AHF.

Test Result Variable(s): Multimarker Model				
Area	Std. Error	*p*-Value	95% Confidence Interval	
			Lower Bound	Upper Bound
0.819	0.074	0.006	0.675	0.964

**Table 10:** Performance of the multimarker model (NT-proBNP, GDF-15, age, GFR, LVEF) for prediction of 1-year mortality.

Parameter	NT-proBNP ≥ 5304 pg/mL	NT-proBNP < 5304 pg/mL
Omnibus test (χ^2^, df, *p*)	12.718 (5), *p* = 0.026	7.299 (5), *p* = 0.199
Model significance	Significant	Not significant
Nagelkerke R^2^	0.472	0.306
Cox & Snell R^2^	0.346	0.216
−2 Log Likelihood	26.712	29.353
Hosmer–Lemeshow χ^2^ (df, *p*)	4.520 (8), *p* = 0.807	14.164 (8), *p* = 0.073
Model calibration	Excellent	Acceptable
Most influential variables (borderline *p*-values)	GDF-15 (*p* ≈ 0.07–0.09), Age (*p* = 0.049)	GDF-15 (*p* = 0.09, NS)
Explained variance of 1-year mortality	47.2%	30.6%

## Data Availability

The original contributions presented in this study are included in the article. Further inquiries can be directed to the corresponding author.

## Supplementary Materials

The following supporting information can be downloaded at https://www.mdpi.com/article/10.3390/life16030503/s1, Table S1: Baseline laboratory parameters; Table S2: Correlations between GDF-15 levels and relevant parameters; Figure S1: ROC curve for the relationship between cardiac biomarkers and 3-month mortality rate; Table S3: Detailed analysis of the AUC for cardiac biomarkers and 3-month mortality; Figure S2: ROC curve for the relationship between cardiac biomarkers and 6-month mortality rate; Table S4: Detailed analysis of the AUC for cardiac biomarkers and 6-month mortality; Figure S3: ROC curve for the relationship between cardiac biomarkers and 1-year mortality rate; Table S5: Detailed analysis of the AUC for cardiac biomarkers and 1-year mortality.

## References

[1] Arrigo M., Jessup M., Mullens W., Reza N., Shah A.M., Sliwa K., Mebazaa A. (2020). Acute heart failure. Nat. Rev. Dis. Primers.

[2] Younis A., Mulla W., Goldkorn R., Klempfner R., Peled Y., Arad M., Freimark D., Goldenberg I. (2019). Differences in Mortality of New-Onset (De-Novo) Acute Heart Failure Versus Acute Decompensated Chronic Heart Failure. Am. J. Cardiol..

[3] McDonagh T.A., Metra M., Adamo M., Baumbach A., Böhm M., Burri H., Butler J., Čelutkienė J., Chioncel O., Cleland J.G.F. (2021). 2021 ESC Guidelines for the diagnosis and treatment of acute and chronic heart failure. Eur. Heart J..

[4] Miró Ò., Chioncel O., Mebazaa A., Sato N., Butler J., Davison B., Biegus J., Pagnesi M., Ambrosy A.P., Savarese G. (2025). Early diagnosis and treatment of acute heart failure in prehospital and emergency settings. Part 1 of the International Expert Opinion Series on acute heart failure management. Eur. J. Emerg. Med. Off. J. Eur. Soc. Emerg. Med..

[5] Wettersten N. (2021). Biomarkers in Acute Heart Failure: Diagnosis, Prognosis, and Treatment. Int. J. Heart Fail..

[6] Mauro C., Chianese S., Cocchia R., Arcopinto M., Auciello S., Capone V., Carafa M., Carbone A., Caruso G., Castaldo R. (2023). Acute heart failure (AHF) manifests through a combination of symptoms and clinical signs, largely driven by pulmonary and systemic congestion. Patients commonly experience dyspnea at rest or with exertion, orthopnea, fatigue, and reduced exercise toleranc. J. Clin. Med..

[7] Wettersten N., Maisel A. (2015). Role of Cardiac Troponin Levels in Acute Heart Failure. Card. Fail. Rev..

[8] Long B., Koyfman A., Gottlieb M. (2019). Diagnosis of Acute Heart Failure in the Emergency Department: An Evidence-Based Review. West J. Emerg. Med..

[9] Pourafkari L., Tajlil A., Nader N.D. (2019). Biomarkers in diagnosing and treatment of acute heart failure. Biomark Med..

[10] Maisel A.S., Choudhary R. (2012). Biomarkers in acute heart failure-state of the art. Nat. Rev. Cardiol..

[11] Mallick A., Januzzi J.L. (2015). Biomarcadores en la insuficiencia cardiaca aguda. Rev. Esp. Cardiol..

[12] Miftode R.S., Constantinescu D., Cianga C.M., Petris A.O., Costache I.I., Mitu O., Miftode I.-L., Mitu I., Timpau A.-S., Duca S.-T. (2022). A Rising Star of the Multimarker Panel: Growth Differentiation Factor-15 Levels Are an Independent Predictor of Mortality in Acute Heart Failure Patients Admitted to an Emergency Clinical Hospital from Eastern Europe. Life.

[13] Dmour B.A., Badescu M.C., Tuchiluș C., Cianga C.M., Constantinescu D., Dima N., Duca Ș.T., Dmour A., Costache A.D., Cepoi M.-R. (2025). Can Endothelin-1 Help Address the Diagnostic and Prognostic Challenges in Multimorbid Acute Heart Failure Patients?. Life.

[14] Dmour B.A., Costache A.D., Dmour A., Huzum B., Duca Ștefania T., Chetran A., Miftode R.Ș., Afrăsânie I., Tuchiluș C., Cianga C.M. (2023). Could Endothelin-1 Be a Promising Neurohormonal Biomarker in Acute Heart Failure?. Diagnostics.

[15] Castiglione V., Aimo A., Vergaro G., Saccaro L., Passino C., Emdin M. (2021). Biomarkers for the diagnosis and management of heart failure. Heart. Fail. Rev..

[16] Eddy A.C., Trask A.J. (2021). Growth differentiation factor-15 and its role in diabetes and cardiovascular disease. Cytokine Growth Factor Rev..

[17] Meijers W.C., Bayes-Genis A., Mebazaa A., Bauersachs J., Cleland J.G.F., Coats A.J.S., Januzzi J.L., Maisel A.S., McDonald K., Mueller T. (2021). Circulating heart failure biomarkers beyond natriuretic peptides: Review from the Biomarker Study Group of the Heart Failure Association (HFA), European Society of Cardiology (ESC). Eur. J. Heart Fail..

[18] Lawton L.N., De Fatima Bonaldo M., Jelenc P.C., Qiu L., Baumes S.A., Marcelino R.A., de Jesus G.M., Wellington S., A Knowles J., Warburton D. (1997). Identification of a novel member of the TGF-beta superfamily highly expressed in human placenta. Gene.

[19] Rochette L., Dogon G., Zeller M., Cottin Y., Vergely C. (2021). Gdf15 and cardiac cells: Current concepts and new insights. Int. J. Mol. Sci..

[20] Arkoumani M., Papadopoulou-Marketou N., Nicolaides N.C., Kanaka-Gantenbein C., Tentolouris N., Papassotiriou I. (2020). The clinical impact of growth differentiation factor-15 in heart disease: A 2019 update. Crit. Rev. Clin. Lab. Sci..

[21] Wesseling M., de Poel J.H.C., de Jager S.C.A. (2020). Growth differentiation factor 15 in adverse cardiac remodelling: From biomarker to causal player. ESC Heart Fail..

[22] Kempf T., Wollert K.C. (2009). Growth-Differentiation Factor-15 in Heart Failure. Heart Fail. Clin..

[23] Stahrenberg R., Edelmann F., Mende M., Kockskämper A., Düngen H.D., Lüers C., Binder L., Herrmann-Lingen C., Gelbrich G., Hasenfuß G. (2010). The novel biomarker growth differentiation factor 15 in heart failure with normal ejection fraction. Eur. J. Heart Fail..

[24] Wang Y., Zhen C., Wang R., Wang G. (2019). Growth-differentiation factor-15 predicts adverse cardiac events in patients with acute coronary syndrome: A meta-analysis. Am. J. Emerg. Med..

[25] Corre J., Hébraud B., Bourin P. (2013). Concise Review: Growth Differentiation Factor 15 in Pathology: A Clinical Role?. Stem Cells Transl. Med..

[26] Mueller T., Leitner I., Egger M., Haltmayer M., Dieplinger B. (2015). Association of the biomarkers soluble ST2, galectin-3 and growth-differentiation factor-15 with heart failure and other non-cardiac diseases. Clin. Chim. Acta.

[27] Lok D.J., Klip I.T., Lok S.I., De La Porte P.W.B.A., Badings E., Van Wijngaarden J., Voors A.A., de Boer R.A., van Veldhuisen D.J., van der Meer P. (2013). Incremental prognostic power of novel biomarkers (growth-differentiation factor-15, high-sensitivity C-reactive protein, galectin-3, and high-sensitivity troponin-T) in patients with advanced chronic heart failure. Am. J. Cardiol..

[28] Kosum P., Siranart N., Mattanapojanat N., Phutinart S., Kongruttanachok N., Sinphurmsukskul S., Siwamogsatham S., Puwanant S., Ariyachaipanich A. (2024). GDF-15: A novel biomarker of heart failure predicts short-term and long-term heart-failure rehospitalization and short-term mortality in patients with acute heart failure syndrome. BMC Cardiovasc. Disord..

[29] George M., Jena A., Srivatsan V., Muthukumar R., Dhandapani V. (2016). GDF 15—A Novel Biomarker in the Offing for Heart Failure. Curr. Cardiol. Rev..

[30] Mentz R.J., O’Connor C.M. (2016). Pathophysiology and clinical evaluation of acute heart failure. Nat. Rev. Cardiol..

[31] King M., Kingery J., Casey B. (2012). Diagnosis and Evaluation of Heart Failure. Am. Fam. Physician.

[32] Profire BȘtefania Lupașcu F.G., Stătescu C., Șorodoc V., Sascău R.A., Profire L., Șorodoc L. (2025). Heart Failure Biomarkers—Pathophysiology, Diagnosis, Prognosis and Clinical Relevance. Int. J. Mol. Sci..

[33] di Candia A.M., de Avila D.X., Moreira G.R., Villacorta H., Maisel A.S. (2021). Growth differentiation factor-15, a novel systemic biomarker of oxidative stress, inflammation, and cellular aging: Potential role in cardiovascular diseases. Am. Heart J. Plus: Cardiol. Res. Pract..

[34] Kempf T., von Haehling S., Peter T., Allhoff T., Cicoira M., Doehner W., Ponikowski P., Filippatos G.S., Rozentryt P., Drexler H. (2007). Prognostic Utility of Growth Differentiation Factor-15 in Patients with Chronic Heart Failure. J. Am. Coll. Cardiol..

[35] Wang F., Guo Y., Yu H., Zheng L., Mi L., Gao W. (2010). Growth differentiation factor 15 in different stages of heart failure: Potential screening implications. Biomarkers.

[36] Chan M.M.Y., Santhanakrishnan R., Chong J.P.C., Chen Z., Tai B.C., Liew O.W., Ng T.P., Ling L.H., Sim D., Leong K.T.G. (2016). Growth differentiation factor 15 in heart failure with preserved vs. reduced ejection fraction. Eur. J. Heart Fail..

[37] Ha G., De Torres F., Arouche N., Benzoubir N., Ferratge S., Hatem E., Anginot A., Uzan G. (2019). GDF15 secreted by senescent endothelial cells improves vascular progenitor cell functions. PLoS ONE.

[38] Alem M.M. (2019). Endothelial dysfunction in chronic heart failure: Assessment, findings, significance, and potential therapeutic targets. Int. J. Mol. Sci..

[39] Mazagova M., Buikema H., Landheer S.W., Vavrinec P., Van Buiten A., Henning R.H., Deelman L.E. (2013). Growth differentiation factor 15 impairs aortic contractile and relaxing function through altered caveolar signaling of the endothelium. Am. J. Physiol. Heart Circ. Physiol..

[40] Tang Y., Liu T., Sun S., Peng Y., Huang X., Wang S., Zhou Z. (2024). Role and Mechanism of Growth Differentiation Factor 15 in Chronic Kidney Disease. J. Inflamm. Res..

[41] Lasaad S., Crambert G. (2024). GDF15, an Emerging Player in Renal Physiology and Pathophysiology. Int. J. Mol. Sci..

[42] Fukuda T., Yazawa H., Nishikawa R., Tokoi S., Kayashima R., Kono K., Sakuma M., Abe S., Toyoda S., Nakajima T. (2024). Physiological Role of Serum Growth Differentiation Factor-15 (GDF-15) Level and Iron Metabolism in Community-Dwelling Older Adults. Cureus.

[43] Wollert K.C., Kempf T. (2012). Growth differentiation factor 15 in heart failure: An update. Curr. Heart Fail. Rep..

[44] May B.M., Pimentel M., Zimerman L.I., Rohde L.E. (2021). GDF-15 as a biomarker in cardiovascular disease. Arq. Bras. Cardiol..

[45] Nyárády B.B., Kiss L.Z., Bagyura Z., Merkely B., Dósa E., Láng O., Kőhidai L., Pállinger É. (2024). Growth and differentiation factor-15: A link between inflammaging and cardiovascular disease. Biomed. Pharmacother..

[46] Hao J., Cheang I., Zhang L., Wang K., Wang H.M., Wu Q.Y., Zhou Y.-L., Zhou F., Xu D.-J., Zhang H.-F. (2019). Growth differentiation factor-15 combined with N-terminal prohormone of brain natriuretic peptide increase 1-year prognosis prediction value for patients with acute heart failure: A prospective cohort study. Chin. Med. J..

[47] Álvarez-García J., García-Osuna Á., Vives-Borrás M., Ferrero-Gregori A., Martínez-Sellés M., Vázquez R., González-Juanatey J.R., Rivera M., Segovia J., Pascual-Figal D. (2021). A 3-Biomarker 2-Point-Based Risk Stratification Strategy in Acute Heart Failure. Front Physiol..

[48] Bouabdallaoui N., Claggett B., Zile M.R., McMurray J.J.V., O’Meara E., Packer M., Prescott M.F., Swedberg K., Solomon S.D., Rouleau J.L. (2018). Growth differentiation factor-15 is not modified by sacubitril/valsartan and is an independent marker of risk in patients with heart failure and reduced ejection fraction: The PARADIGM-HF trial. Eur. J. Heart Fail..

