# Supplementary material for: Integrating GDF-15 into Multimarker Assessment of Acute Heart Failure: Diagnostic and Prognostic Implications
Source: Life (Basel). 2026 Mar 19;16(3):503. doi: 10.3390/life16030503 (PMC13027845; doi:10.3390/life16030503)
Supplement: Supplementary file 1 [file life-16-00503-s001.zip › life-4170423-supplementary.pdf]

Supplementary Table S1. Baseline laboratory parameters

Parameter	Total (n=102) Mean ± Std. Dev	AHF (n=60) Mean ± Std. Dev	Control Group (n=42) Mean ± Std. Dev	*p*-Value
**NT-proBNP (pg/mL)**	4413± 5870	7282 ± 6209	315 ± 389	<0.001
**hs-cTnI (ng/L)**	2972±10967	5046± 13974	8.74± 17.99	0.007
**GDF-15 (pg/mL)**	591 ± 491	796 ± 545	298 ± 130	<0.001
**ET-1 (pg/mL)**	3.49± 6.46	4.99 ± 8.12	1.36 ± 0.44	0.001
**CK (mg/dL)**	286 ± 861	397 ± 1103	128 ± 172	0.068
**CK-MB(mg/dL**	43.38± 112	55.98 ± 142	24.95 ± 34.89	0.110
**CRP (mg/dL)**	2.2± 3.17	2.72 ±3.45	1.46 ± 2.58	0.039
**Leukocyte (cmm)**	8306±2363	8722 ± 2627	7712 ± 1791	0.023
**Hemoglobin (g/dL)**	13.35±2.05	13.05 ± 2.23	13.77 ±1.7	0.083
**Glucose (mg/dL)**	121± 48.14	127 ± 51.56	111±41.67	0.106
**Sodium (mmol/L)**	139 ± 4.16	138 ±4.91	140 ± 2.6	0.059
**Potassium (mmol/L)**	4.46 ± 4.2	4.54 ± 0.65	4.34± 0.5	0.104
**Alkaline reserve (mEq/L)**	23.87 ± 3.62	23.24 ± 4.04	24.76 ± 2.07	0.036
**Urea (mg/dL)**	53.72±26.92	62.1 ± 30.73	41.74± 13.27	<0.001
**Creatinine (mg/dL)**	1.12 ± 0.46	1.24 ± 0.51	0.95 ± 0.3	0.001
**GFR (Cockcroft Gault) mL/min/1.73 m²**	73.75±33.6	69.85 ± 33.29	84.88± 32.77	0.078
**ALT (IU/L)**	52.57±93.1	71.03±117.75	26.19±14.44	0.005
**AST (IU/L)**	47.56±80.0	59.33±97.26	30.74± 41.11	0.045
**GGT (IU/L)**	53.29±52.5	63.8 ± 58.34	37.9 ± 38.39	0.008
**LDH (U/L)**	273 ± 277	329 ± 347	188 ± 36.2	0.003
**TSH (mIU/L)**	1.96 ± 1.5	2.22 ± 1.62	1.62 ± 1.26	0.080
**Total cholesterol (mg/dL)**	159 ± 47.55	142 ± 42.19	182 ± 45.3	<0.001
**LDL cholesterol (mg/dL)**	107 ± 43.96	96.22±37.42	124 ± 47.59	0.001
**HDL cholesterol (mg/dL)**	39.93±14.25	36.22±14.75	45.24 ± 11.75	0.001
**Triglycerides(mg/dL)**	117 ± 62.04	107 ± 44.09	130 ± 79.82	0.067
**Iron (μg/dL)**	64.46 ± 34.4	53.28±29.27	80.43 ± 35.38	<0.001
**Ferritin (ng/mL)**	203 ± 260	215± 315	186 ± 153	0.586

Legend: AHF—acute heart failure; GDF-15—growth differentiating factor-15; NT-proBNP—amino-terminal pro-B-type natriuretic peptide; hs-cTnI—high-sensitivity cardiac troponin I; ET-1—endothelin-1; CRP—reactive protein; LDH—lactate dehydrogenase; CK—creatine kinase; CK-MB—creatine kinase myocardial band; ALT—alanine transaminase; AST—aspartate transaminase; LDL—low-density lipoprotein; HDL—high-density lipoprotein cholesterol; TSH— thyroid-stimulating hormone; GFR—estimated glomerular filtration rate.

Supplementary Table S2. Correlations between GDF-15 levels and relevant parameters.

GDF-15	p-value	R
**Age**	0.01	0.32
**Systolic blood pressure**	0.45	−0.09
**Diastolic blood pressure**	0.48	-0.09
**Heart rate**	0.97	0.01
**SpO2**	0.39	-0.11
**LV end-diastolic diameter**	0.04	-0.25
**Right ventricle diameter**	0.40	-0.1
**LVEF**	0.67	-0.05
**TAPSE**	<0.01	−0.35
**NT-proBNP**	0.01	0.32
**hs-cTnI**	0.54	0.31
**LDH**	<0.01	0.36
**CRP**	0.11	0.2
**ET-1**	<0.01	0.39
**Hemoglobin**	<0.01	-0.34
**Sodium**	0.05	−0.25
**Potassium**	<0.01	0.34
**Alkaline reserve**	0.01	−0.3
**GFR**	<0.01	-0.51
**Urea**	0.01	0.3
**Creatinine**	<0.01	0.38
**Total cholesterol**	0.04	−0.26
**LDL cholesterol**	0.18	−0.17
**HDL cholesterol**	0.11	−0.2
**Triglycerides**	0.22	−0.16
**Iron**	<0.01	−0.26
**Ferritin**	0.84	0.02

Legend: LV—left ventricle; GDF-15—growth differentiating factor-15; ET-1—endothelin-1; NT-proBNP—amino-terminal pro-B-type natriuretic peptide; hs-cTnI—high-sensitivity cardiac troponin I; CRP—reactive

protein; LDH—lactate dehydrogenase; CK—creatine kinase; CK-MB—creatine kinase myocardial band; ALT—alanine transaminase; AST—aspartate transaminase; LDL—low-density lipoprotein; HDL—high-density lipoprotein cholesterol; TSH—thyroid-stimulating hormone; LVEF—left ventricular ejection fraction; TAPSE—tricuspid annular plane systolic excursion.

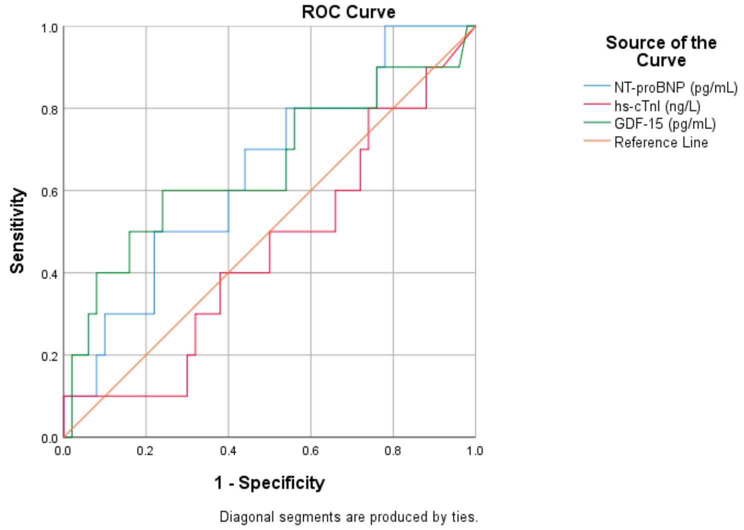

Supplementary Figure S1. ROC curve for the relationship between cardiac biomarkers and 3-month mortality rate

Supplementary Table S3. Detailed analysis of the AUC: cardiac biomarkers and 3-month mortality rate**.**

Test Result Variable	Area	Std. Error	p-Value	95% Confidence Interval	
				Lower Bound	Upper Bound
NT-proBNP, pg/mL	.646	.093	.148	.463	.829
hs-cTnI, ng/L	.454	.100	.648	.258	.650
GDF-15, pg/mL	.659	.108	.115	.447	.871

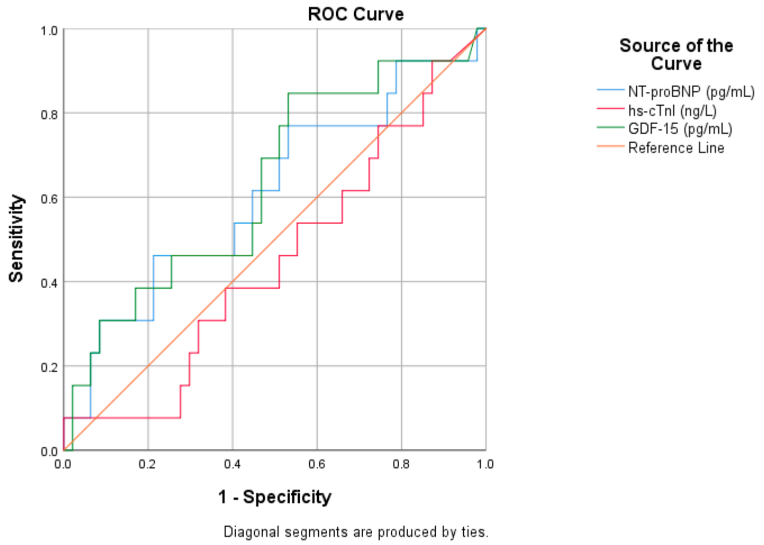

Supplementary Figure S2. ROC curve for the relationship between cardiac biomarkers and 6-month mortality rate

Supplementary Table S4. Detailed analysis of the AUC: cardiac biomarkers and 6-month mortality rate.

Test Result Variable	Area	Std. Error	p-Value	95% Confidence Interval	
				Lower Bound	Upper Bound
NT-proBNP, pg/mL	.610	.093	.226	.428	.793
hs-cTnI, ng/L	.450	.088	.584	.277	.623
GDF-15, pg/mL	.634	.089	.141	.460	.808

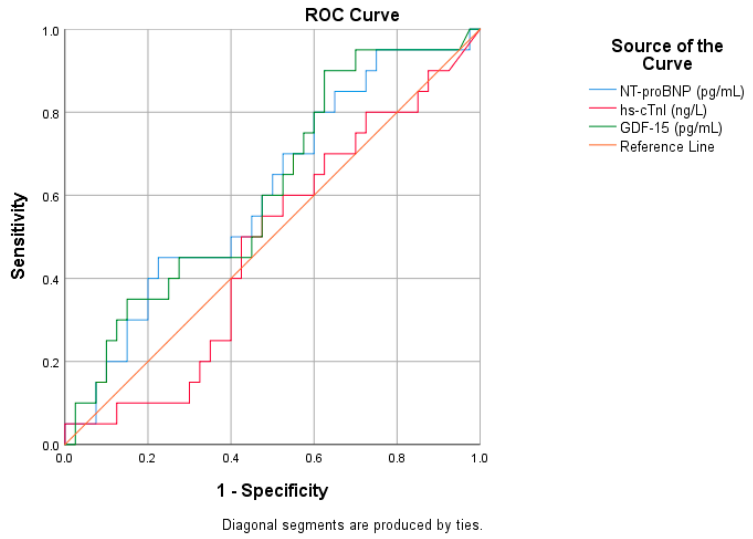

Supplementary Figure S3. ROC curve for the relationship between cardiac biomarkers and 1-year mortality rate

Supplementary Table S5. Detailed analysis of the AUC: cardiac biomarkers and 1-year mortality rate.

Test Result Variable	Area	Std. Error	p-Value	95% Confidence Interval	
				Lower Bound	Upper Bound
NT-proBNP, pg/mL	.609	.076	.172	.459	.759
hs-cTnI, ng/L	.477	.078	.778	.325	.630
GDF-15, pg/mL	.616	.076	.147	.467	.764
